# UNICORN Babies: Understanding Circulating and Cerebral Creatine Levels of the Preterm Infant. An Observational Study Protocol

**DOI:** 10.3389/fphys.2019.00142

**Published:** 2019-03-07

**Authors:** Mary J. Berry, Melissa Schlegel, Greg M. Kowalski, Clinton R. Bruce, Damien L. Callahan, Miranda L. Davies-Tuck, Hayley Dickinson, Angus Goodson, Angie Slocombe, Rod J. Snow, David W. Walker, Stacey J. Ellery

**Affiliations:** ^1^ Department of Paediatrics and Child Health, University of Otago, Wellington, New Zealand; ^2^ Capital and Coast District Health Board, Wellington, New Zealand; ^3^ School of Exercise Sciences, Institute for Physical Activity and Nutrition, Deakin University, Geelong, VIC, Australia; ^4^ Centre for Cellular and Molecular Biology, School of Life and Environmental Science, Deakin University, Melbourne, VIC, Australia; ^5^ The Ritchie Centre, Hudson Institute of Medical Research, and Department of Obstetrics and Gynaecology, Monash University, Clayton, VIC, Australia; ^6^ School of Health and Biomedical Sciences, RMIT University, Melbourne, VIC, Australia

**Keywords:** premature, nutrition, brain metabolism, cellular energy, neurodevelopment

## Abstract

Creatine is an essential metabolite for brain function, with a fundamental role in cellular (ATP) energy homeostasis. It is hypothesized that preterm infants will become creatine deplete in the early postnatal period, due to premature delivery from a maternal source of creatine and a limited supply of creatine in newborn nutrition. This potential alteration to brain metabolism may contribute to, or compound, poor neurological outcomes in this high-risk population. Understanding Creatine for Neurological Health in Babies (UNICORN) is an observational study of circulating and cerebral creatine levels in preterm infants. We will recruit preterm infants at gestational ages 23^+0^–26^+6^, 27^+0^–29^+6^, 30^+0^–32^+6^, 33^+0^–36^+6^, and a term reference group at 39^+0^–40^+6^ weeks of gestation, with 20 infants in each gestational age group. At birth, a maternal capillary blood sample, as well as a venous cord blood sample, will be collected. For preterm infants, serial infant plasma (heel prick), urine, and nutrition samples [total parenteral nutrition (TPN), breast milk, or formula] will be collected between birth and term “due date.” Key fetomaternal information, including demographics, smoking status, and maternal diet, will also be collected. At term corrected postnatal age (CPA), each infant will undergo an MRI/^1^H-MRS scan to evaluate brain structure and measure cerebral creatine content. A general movements assessment (GMA) will also be conducted. At 3 months of CPA, infants will undergo a second GMA as well as further neurodevelopmental evaluation using the Developmental Assessment of Young Children – Second Edition (DAYC-2) assessment tool. The primary outcome measures for this study are cerebral creatine content at CPA and plasma and urine creatine and guanidinoacetate (creatine precursor) concentrations in the early postnatal period. We will also determine associations between (1) creatine levels at term CPA and neurodevelopmental outcomes (MRI, GMA, and DAY-C); (2) dietary creatine intake and circulating and cerebral creatine content; and (3) creatine levels and maternal characteristics. Novel approaches are needed to try and improve preterm-associated brain injury. Inclusion of creatine in preterm nutrition may better support *ex utero* brain development through improved cerebral cellular energy availability during a period of significant brain growth and development.

**Ethics Ref:** HDEC 18/CEN/7 New Zealand.

**ACTRN:** ACTRN12618000871246.

## Introduction

The global incidence of preterm birth (delivery prior to 37 completed weeks of gestation) is reported to be ~11% (Blencowe et al., 2013). Advances in perinatal care mean that increasing numbers of infants born even at extremes of gestational age (extreme preterm: <28 weeks of gestational age) survive beyond the neonatal period (Davidoff et al., 2006; Mohangoo et al., 2011; Lawn et al., 2013). Additionally, within resource-rich neonatal care settings, the age of human viability continues to fall with increasing survival rates at even 22 and 23 weeks of gestational age. However, this increase in survival has not been coupled with a reduction in long-term morbidity. Neurodevelopmental disorders, including those of executive function, psychiatric, and behavioral sequelae (Blencowe et al., 2013), are more prevalent in those born preterm than at term, with increasing risk, the lesser the gestational age at birth (Crump et al., 2010; Johnson et al., 2010).

The etiology of brain injury in preterm infants is complex and often unexplained. Historically, intraventricular hemorrhage (IVH) with resultant disruption of brain tissue and formation of macrocystic lesions was associated with global developmental delay. With the improvements in perinatal care, high grade IVH and/or periventricular leukomalacia (PVL) has become far less common, and standard cranial ultrasound or magnetic resonance imaging (MRI) examination usually do not demonstrate gross structural lesions (Penn et al., 2016). In Australia and New Zealand, the rate of high grade IVH in infants born prior to 32 weeks’ gestation declined from 8% to just over 4% in the period between 2004 and 2013 (Chow et al., 2013). As such, longitudinal studies now indicate that a large percentage of children born preterm have neurodevelopmental problems in the absence of structural brain abnormalities detected with conventional imaging (Constable et al., 2008; Mullen et al., 2011; Aeby et al., 2013). These data suggest that conventional prognostication based on measures of cerebral structure is poorly discriminatory and will not always identify infants at risk of neurodevelopmental morbidity. We, and others, propose that our focus should be widened to explore the role of altered brain metabolism in the pathophysiology of preterm brain injury and neurodevelopmental disability (Koob et al., 2016).

Creatine is a metabolite essential for brain development and energy metabolism (Braissant et al., 2007). It is an amino acid derivative that fuels the creatine kinase circuit; a phosphagen system that mitigates temporal imbalances in energy (ATP) supply and demand (Wallimann et al., 1992). *Via* the creatine kinase circuit, creatine and its phosphorylated derivative (phosphocreatine, PCr) shuttle high-energy phosphate groups from the mitochondria into the cytosol, for the regeneration of ATP from ADP (Ellington, 2001). The creatine kinase isoforms required to run the circuit are found in the cerebellum, choroid plexus, hippocampal granular layer, and pyramidal cells of the brain (Hemmer et al., 1994) and are involved in maintaining ATP homeostasis in the central nervous system (CNS) from early in embryonic development through to adulthood (Braissant et al., 2001; Braissant et al., 2007).

Both creatine and phosphocreatine are spontaneously broken down into creatinine at a rate of ~1.7%/day (Brosnan and Brosnan, 2007). Thus, there is a requirement to replenish creatine and PCr stores on a daily basis. Creatine can be obtained from the diet and is also endogenously synthesized *de novo*. Synthesis involves the production of guanidinoacetate (GAA) from arginine and glycine, in a reaction catalyzed by arginine:glycine aminotransferase (AGAT) that occurs predominately in the kidney and pancreas. GAA is then methylated by guanidinoacetate N-methyltransferase (GAMT), mainly in the liver, to produce creatine. Once synthesized or absorbed, creatine is transported into cells *via* the sodium-dependent SLC6A8 creatine transporter (Wyss and Kaddurah-Daouk, 2000). Creatine is able to cross the blood-brain barrier (Ohtsuki et al., 2002), and the brain itself also has some inherent capacity to synthesize creatine, with both AGAT and GAMT expressed by neurons, astrocytes, and oligodendrocytes (Braissant et al., 2001; Tachikawa et al., 2004; Nakashima et al., 2005). Despite the importance of creatine for brain growth and development, the relative importance of cerebral creatine derived from systemic rather than local synthesis has not yet been established.

*In utero* fetal magnetic resonance spectroscopy (^1^H-MRS) studies indicate cerebral creatine accretion occurs from 18 to 40 weeks of gestation (Evangelou et al., 2015). Postnatal studies have also clearly identified that an increase in cerebral creatine concentration occurs during the first 3 months of life (Blüml et al., 2012; Blüml et al., 2014). The importance of a maternal source of creatine for the developing fetus and the continued postnatal accretion of cerebral creatine is made evident by those infants diagnosed with inherited creatine deficiency syndromes. These infants typically raise no concerns antenatally; however, as postnatal creatine levels fall, they become progressively more symptomatic with altered neurological state (Almeida et al., 2006) and complex neurological symptoms including impaired psychomotor development, seizures, and cognitive impairment (Battini et al., 2006).

In adults, creatine is acquired *via* the diet and *de novo* synthesis, with each contributing ~50% (Brosnan and Brosnan, 2007). However, in infancy, due to low levels of creatine in human breast milk and commercial formulas, a term infant most likely synthesizes between 64 and 93% of their daily creatine requirements (Edison et al., 2013). The age at which a fetus/newborn develops the capacity to synthesize creatine is currently unknown, but it requires sufficient renal, hepatic, and cerebral maturity to express the enzymes necessary for creatine synthesis. Evaluation of AGAT and GAMT expression in the rat embryo found that AGAT was expressed in isolated cells of the CNS from 0.6 gestation; however, widespread expression of AGAT and rudimentary GAMT expression were not apparent before 0.8 gestation (Braissant et al., 2005). Our own studies in the precocial spiny mouse found that the fetal kidney and liver do not express AGAT and GAMT before 0.9 gestation, an age and maturation equivalent to ~35 weeks’ human gestation (Ireland et al., 2009). Taken together, these observations raise compelling questions about progressive creatine depletion in preterm infants, and whether this complication of nutritional compromise predisposes or compounds their risk of altered cerebral metabolism and thus long-term neurodevelopmental disability.

To date, a number of small observational studies have identified perturbations in creatine homeostasis in preterm infants (Blüml et al., 2014; Koob et al., 2016). Koob et al. (2016) reported that at term CPA creatine concentrations in the centrum semiovale of infants born preterm (born 29.1 ± 2 weeks) were reduced compared to those born at term (Koob et al., 2016). In addition, Lage et al. (2013) found that by hospital discharge, preterm infants had higher urinary GAA and reduced urinary creatine excretion, suggesting differences in systemic creatine synthesizing capacity. This was particularly apparent in their very preterm group (28–29 weeks) (Lage et al., 2013).

No studies have characterized longitudinal changes in creatine metabolism among preterm infants or the association between cerebral creatine content and neurodevelopmental outcomes. Further to this, no studies have monitored systemic creatine levels (both circulating and excreted) in a single preterm population nor have they assessed nutritional creatine availability for preterm infants fed with total parenteral nutrition (TPN), preterm infant formulas, or breast milk.

We hypothesize that following preterm birth, peripheral and cerebral creatine levels will fall, so that at term CPA, preterm infants will have relative systemic and cerebral creatine deficiency. Furthermore, this reduction in creatine bioavailability will jeopardize normal brain metabolism and development, ultimately predisposing the infant to neurodevelopmental dysfunction.

The overall aim of this study is to assess whether preterm infants become creatine deplete in the early postnatal period, by measuring the preterm infants’ circulating and excreted levels of creatine and GAA and comparing these measures to cerebral creatine content, as determined by ^1^H-MRS, at term corrected age. This study will correlate preterm creatine levels with neurological outcomes using MRI, general movements assessment (GMA), and the Developmental Assessment of Young Children – Second Edition (DAYC-2) Test (Novak et al., 2017). This study will also establish the levels of creatine provided through dietary intake during the days and weeks after preterm birth. Results of this study may support the use of creatine supplementation as standard nutritional care of the preterm infant, in order to improve neurodevelopmental function in this vulnerable population.

### Specific Aims

1. Compare cerebral, plasma, and urine creatine and GAA content between preterm and term infants at CPA.
2. Describe preterm infant plasma and urine creatine and GAA concentrations and creatine:GAA ratio in the early postnatal period.
3. Describe associations between cerebral creatine content, plasma, and urine creatine concentrations with maternal and infant characteristics at CPA.
4. Compare creatine, GAA, arginine, glycine, and methionine concentrations in standard preterm nutrition, including TPN, preterm infant formulas, and breast milk.
5. Determine whether dietary source of creatine (TPN, formula, or breast milk) is related to plasma or urine creatine concentrations in the early postnatal period.
6. Describe associations between cerebral creatine content and blood and urine creatine concentrations with neurological outcomes (GMA, MRI, and DAYC-2) at CPA and at 3 months of corrected postnatal age.

## Method/Design

### Study Design

Prospective, single-center, observational study.

### Study Setting

Neonatal Intensive Care Unit and Postnatal Wards, Capital Coast District Hospital Board (CCDHB), Wellington, New Zealand.

### Primary Outcome Measures

1. Cerebral creatine content at term CPA
2. Plasma (bi-weekly) and urine (weekly) creatine and GAA levels from birth until hospital discharge and at CPA
3. Creatine, GAA, and amino acid levels in infant nutrition (TPN, formula, or breast milk) supplied from birth until discharge and at CPA

### Secondary Outcome Measures

1. Global injury score from MRI at term CPA
2. General movement assessment score at term CPA and 3 months of CPA
3. DAYC-2 assessment score at 3 months of CPA

### Study Population

#### Inclusion Criteria

1. Preterm infants receiving care within the Neonatal Intensive Care Unit at CCDHB or healthy term infants at 39–40 weeks’ gestation from low-risk pregnancies delivered at CCDHB.

#### Exclusion Criteria

1. Babies with major congenital, genetic, or chromosomal abnormalities known to affect neurodevelopmental outcomes
2. Hypoxic ischemic encephalopathy
3. Inborn (proven or suspected) errors of metabolism
4. Fetal growth restriction (birth weight < 10th percentile on customized percentiles)

### Recruitment

We will recruit infants at gestational ages 23^+0^–26^+6^, 27^+0^–29^+6^, 30^+0^–32^+6^, 33^+0^–36^+6^, and 39^+0^–40^+6^ weeks. We will aim to capture 20 infants at each time point (with equal numbers of males and females). Eligible infants will be identified based on gestational age at delivery and consideration of inclusion and exclusion criteria. Pregnant women will be approached at presentation to Delivery Suite to discuss study participation. If consent is obtained, the mother and infant will be recruited to the study and the consulting obstetric, midwifery, neonatal, and nursing team advised of their involvement. Where pre-delivery consent is not obtained due to rapidity of labor and/or other clinical factors, cord blood will be collected preemptively, and retrospective consent and maternal blood sample sought within 24 h of delivery. If the parents do not wish to participate, the cord blood sample will be disposed of using routine clinical biological waste disposal systems and no further study samples collected.

### Sample and Data Collection

#### Blood, Urine, and Nutrition Sampling

**Figure 1:**
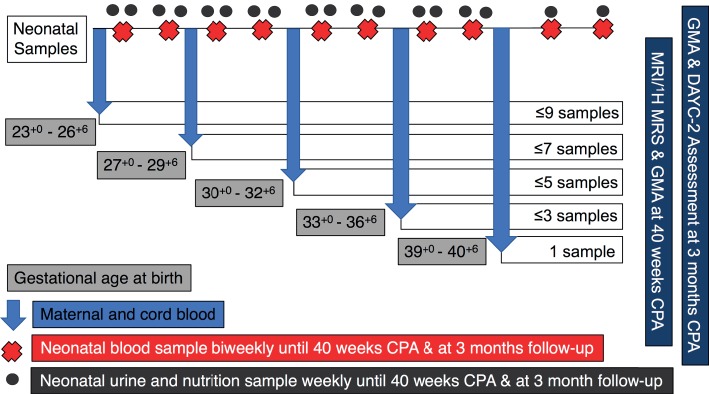
Schematic representation of UNICORN sampling regime. Samples of neonatal blood, urine, and nutrition will be collected weekly or bi-weekly for the duration of the infants stay in hospital. Magnetic resonance imaging (MRI) and magnetic resonance spectroscopy (^1^H-MRS) will be performed at term corrected postnatal age (CPA). Infants will also undergo a general movements assessment (GMA) at CPA and at 3 months CPA. At the follow-up appointment at 3 months CPA, further neurological assessment will be completed using the Developmental Assessment of Young Children – Second Edition (DAYC-2) assessment tool.

A schematic overview of the sampling regime for each participant is outlined in Figure 1. At the time of birth, we will use a finger prick to collect ~120 μl of maternal whole blood using a capillary tube. A 2-ml venous cord blood sample will also be collected from which a ~120 μl sample will be obtained using a capillary tube. Then, while infants are receiving care in the NICU, or on postnatal wards, and at follow-up appointments, the following samples will be collected. Sample frequency will be dictated by gestational age at birth.

**Blood sampling**: A blood sample will be collected biweekly. We will perform a heel prick to collect ~120 μl of whole blood using a capillary tube. All blood samples (cord, maternal, or infant) will be transferred to a Hemaspot™ SE blood separation device to facilitate collection of the cell-free blood fraction (referred to throughout as plasma for clarity) from a small volume of whole blood. Hemaspots are allowed to dry at room temperature before being stored at −20°C for future analysis.

**Urine sampling**: Around the same time as the blood collection, and on each alternate week, we will place some gauze into the infant’s nappy to facilitate a spot urine collection. Once obtained, this sample will be transferred to an Eppendorf tube and stored at −80°C for future analysis.

**Nutrition sampling**: A 200 μl sample of the nutrition (TNP, formula, and breast milk) being fed to each infant at the time of the blood and urine sampling will be collected. If in powdered form (formula), this will be reconstituted following standard practices. All samples will be transferred into an Eppendorf tube and stored at −80°C for future analysis.

### Monitoring and Data Collection

The following covariates/potential confounders will be collected as part of this study:

Maternal characteristics and birth outcomes data:

- Maternal age
- Comorbidities/factors (preeclampsia, gestational diabetes, chorioamnionitis, IVF)
- Country of birth
- Dietary preferences (omnivorous, vegetarian, vegan)
- Smoking status
- Parity
- Gravity
- Mode of delivery
- Placental weight

Infant characteristics:

- Gestational age
- Sex
- Birth weight
- Ethnicity
- Weight and corrected postnatal age at the time of each sample collection
- Presence of postnatal infection (blood culture positive and/or treatment with systemic antibiotics for >24 h) or necrotizing enterocolitis (Modified Bells Classification Grade II-III determined by the treating Pediatric Surgeon at time of diagnosis).
- Feeding schedule prior to each sample collection (<24 h)
- Venous hemoglobin and hematocrit count prior to blood sample collection

#### MRI/^1^H-MRS Data Acquisition

At 40 weeks of corrected postnatal age (CPA), each participant will undergo a MRI/^1^H-MRS scan. MR examinations will be performed on a Siemens 3T Skyra using a 16-channel dedicated pediatric head coil. Infants will be examined without sedation using a “feed and wrap” technique according to standard institutional practice. In brief, infants are brought to the MRI unit, fed, and swaddled comfortably while being monitored with continuous pulse oximetry before being placed in the MRI scanner. Standard, size appropriate, hearing protection will be used for all infants. Sequences will be performed to demonstrate brain anatomy and will include T1 3D 1 mm volumetric, T2 1 mm 3D volumetric scans, T2 TSE FLAIR, and T1SE MTC sequences. These sequences will also contribute to the analysis of any detected brain pathology. T2 SWI axial images will be performed to show calcification and blood products. Sequences for the detection of vascular injury and infarction will include diffusion sequences (b value = 2000), resting BOLD, and arterial spin labeling. ^1^H-MRS will provide chemical spectra of the brain to further analyze brain pathology and measurement of cerebral creatine content. For MR spectroscopy, the voxel will be placed over the white matter underlying the central sulcus within the centrum semiovale and spectra acquired with a long echo time (TE 135 ms) (Koob et al., 2016).

#### Neurodevelopmental Observations

At term CPA, prior to the MRI/^1^H-MRS scan, an experienced neurodevelopmental therapist will record a 5-minute video of the infant to conduct a general movements assessment (GMA); a validated tool to noninvasively identify neurological issues, which may lead to cerebral palsy and other developmental disabilities (Spittle et al., 2018). Infants are placed in a supine position, with minimal clothing to allow freedom of movement. The video will be taken when the infant is fully alert, but not directly interacting with parents, healthcare providers, or receiving stimulus from any external sources.

At 3 months of CPA, we will request participants meet again with a developmental therapist. At this appointment, information about general health (including weight gain trajectories, feeding habits, and number of visits to a GP) will be recorded. A questionnaire about developmental milestones at 3 months of age (developed by the American Academy of Pediatrics) will be completed, as well as a Developmental Assessment of Young Children – Second Edition (DAYC-2) Test, a validated tool to identify children with possible developmental delay in the following domains: cognition, communication, social-emotional development, physical development, and adaptive behavior (Saleh and Smadi, 2017). A 5-minute video of the infant will also be recorded to allow for a second GMA to be completed.

### Sample and Data Analysis

#### Creatine, GAA, and Amino Acid Analysis

Creatine, GAA, arginine, glycine, and methionine will be measured in biological and nutrition samples *via* LC-MS/MS, according to the methods of (Tran et al., 2014), with slight modification (Tran et al., 2014). Briefly, a 6-mm hole punch of the “cell-free” plasma fraction of the Hemaspot™, 7 μl of urine, nutrition, or unlabeled standard, will be transferred to 1.5-ml safety lock Eppendorf tubes. 200 μl of methanol containing 10 μM 2,2-d_3_ Creatine (Sigma), 2.5 μM 2,2-d_2_ GAA (Sigma), and 2.5 μM of Labeled Amino Acid Standard Set A (Cambridge Isotope Laboratories Inc.) internal standards is added to each tube. Samples are sonicated for 5 min then vortexed for 20 min, before being centrifuged at 20,000× g for 5 min to pellet any precipitate or filter paper at the bottom of the tube. Supernatant (~170 μl) is transferred into 250-μl glass inserts placed in 2-ml glass vials (Agilent), followed by evaporation to complete dryness in a speed vacuum (Labconco, Kansas, MO, USA). After drying, the GAA and creatine carboxyl groups are derivatized (butylated) by the addition of 100 μl of 3 M butanol-HCL followed by incubation at 60°C for 30 min. After cooling to room temperature, excess butanol-HCL is evaporated under speed vacuum, after which the dry residue is resuspended in 200 μl of methanol: water (1:1 v/v) solution prior to LC-MS/MS analysis. The external standards consist of an eight sample serial dilution series of known concentration range (0–25 μM unlabeled GAA and 0–100 μM unlabeled creatine).

The LC-MS/MS system is composed of a vacuum degasser, binary pumps, column oven, and a temperature-controlled autosampler (Shimadzu, Nexera^®^ UPLC). This will be interfaced with a triple quadrupole mass spectrometer (Shimadzu, LCMS-8030) with electrospray ionization. The LC column (Agilent – 2.1 × 100 mm, 1.8 μm C18 Zorbax Eclipse plus) will be maintained at 30°C and elution carried out with a binary gradient mobile phase at a flow rate of 0.4 ml min^−1^. Mobile phase A will contain water with 0.1% formic acid, and mobile phase B will contain acetonitrile with 0.1% formic acid. The initial mobile phase composition will be 5% B, which is ramped linearly to 50% B over 6 min followed by a 2-min column wash at 100% B, with the column finally undergoing re-equilibration at 5% B for 3 min, thus yielding a total runtime of 11 min. The first 1 min is diverted to waste. The following ESI source conditions will be used: nebulizer gas flow rate 3 L min^−1^, desolvation line temperature at 250°C, heater block temperature 400°C, and drying gas flow rate of 15 L min^−1^. The MS will be operated in positive ionization with multiple reaction monitoring (MRM). The collision energy, Q1 and Q3 pre-bias voltages for the MRM transitions have been optimized using authentic creatine and GAA standards (SIGMA) *via* the automatic optimisation software (Labsolutions, Shimadzu), with optimal collision energy for creatine and GAA for the instrument being −15 eV. The precursor and product ion transitions were creatine 188.2–90 *m/z*, d_3_-Creatine 191.2–93 *m/z,* GAA 174.2–101.1 *m/z*, and d_2_-GAA 176.1–103.1 *m/z*. The sample and standard injection volume will be 5 μl. Peak areas will be determined using Lab solutions post-run browser software (Shimadzu). The creatine, GAA and amino acid concentrations of blood spots, urine, or nutrition samples will be calculated *via* linear regression of the serially diluted external (unlabeled) standard series using the isotope dilution technique.

#### MRI/^1^H-MRS

MRI images will be scored according to the Kidokoro assessment tool (Kidokoro et al., 2013). Briefly, cerebral white matter, cortical gray matter, deep gray matter, and cerebellar abnormalities will be independently graded, and then the scores combined to calculate a single global brain injury score. The assessor will also note the presence of hemorrhage or cystic lesions throughout the brain. Hemorrhagic lesions will be graded using the Papile classification system (Papile et al., 1978) and cystic lesions characterized as periventricular leukomalacia, periventricular hemorrhagic infarction, or other (Barkovich, 2000).

Metabolic ratios from ^1^H-MRS spectra obtained with a TE of 135 ms will be calculated as previously described, with water used as an internal reference to normalize metabolite signal intensities (Koob et al., 2016).

#### Neurodevelopmental Observations

Motor function will be assessed by a developmental therapist trained in GMA and blinded to the clinical history of the infant. At term CPA, infants will be assessed on withering movements, which should include complex movements of the arms, legs, neck, and trunk. Poor withering movements include rigid or chaotic movements that lack fluency and coordination (Spittle et al., 2013) and are predictive of adverse neurodevelopmental outcomes. At 3 months of CPA, infants will be assessed on fidgety movements, which appear as small continual movements of limbs in all directions. Poor quality fidgety movements include larger amplitude and jerking movements (Spittle et al., 2013) and are again predictive of later outcomes. Infants will be scored independently at each postnatal age.

At 3-month CPA, infants’ development will also be reviewed using the DAYC-2 assessment tool (Saleh and Smadi, 2017). An overall score from the five domains tested (cognition, communication, social-emotional development, physical development, and adaptive behavior) will be generated for each infant. Collectively, MRI, GMA, and DAYC-2 scores will be used to assess the potential risk of poor neurological outcomes, based on early diagnosis strategies for cerebral palsy (Novak et al., 2017).

### Sample Size

This study will be the first observational study of creatine homeostasis in the preterm infant that encompasses plasma, urine, nutrition, and cerebral creatine measures across time. As the outcomes are mainly descriptive, no formal power calculation has been performed. However, ^1^H-MRS results reported by Koob et al. (2016) showed comparison of 13 preterm vs term infants allowed for measurement of a statistically significant (−17%) reduction in cerebral creatine content in preterm infants at CPA (tCr/H_2_0 × 10^−3^; term 0.12 ± 0.1 vs preterm 0.10 ± 0.1). This same study had a 20–25% failure rate in obtaining accurate MRI/^1^H-MRS measurements at CPA (Koob et al., 2016). Taking these data into consideration and a potential loss to follow-up at 3-month of 20%, we aim to capture 20 infants in each gestational group.

### Statistical Analysis

Characteristics of the population will be tabulated, along with appropriate descriptive statistics of the study sample. Continuous data will be assessed for normality and expressed as means and standard deviations or median and interquartile range as appropriate. Categorical data will be expressed as counts and proportions. Average creatine content from nutrition per week for each baby in the early postnatal period will be calculated.

Differences in creatine and GAA measures (^1^H-MRS, plasma or urine) between infants born preterm and those born at term will be assessed at CPA using a t-test or Mann-Whitney U test as appropriate. The correlation between gestational age at birth and creatine and GAA measures at CPA will also be determined using a Spearman or Pearson correlation. Preterm infant plasma and urine creatine and GAA concentrations and creatine:GAA ratio in the early postnatal period will be described and plotted. Associations between cerebral creatine content, plasma and urine creatine concentrations with maternal, and infant characteristics at CPA will be determined using Spearman or Pearson correlations for continuous data (maternal age, gestational age, birth weight, parity, gravity, placental weight) or a t-test or Mann-Whitney U test for categorical data (infant sex, mode of delivery, maternal comorbidities, country of birth, ethnicity, and dietary preferences), as appropriate. Differences in creatine, GAA, and amino acid content of nutrition sources (TNP, formula or breast milk) will be determined using a one-way ANOVA or Kruskal-Wallis H test as appropriate. The association between creatine, GAA, and amino acid content of nutrition sources (TNP, formula or breast milk) and infant plasma and urine creatine at CPA will be assessed using a Spearman or Pearson correlation as appropriate. Finally, the association between cerebral creatine content, plasma creatine levels, and the ratio of plasma:cerebral creatine at CPA with GMA, MRI global injury, and DAYC-2 scores will be determined using linear regression at both CPA and at 3 months’ corrected postnatal age. Potential confounders such as gestational age and maternal and infant characteristics will be assessed, along with collinearity, prior to performing multivariable linear regression.

## Discussion

Both creatine and the creatine kinase circuit are critical for brain metabolism (Braissant et al., 2011). It is hypothesized that preterm infants will become creatine deplete in the early postnatal period, due to premature delivery from a maternal source of creatine *in utero,* limited capacity for endogenous synthesis postnatally, and a limited supply of creatine in newborn nutrition. This creatine depletion may alter cerebral creatine metabolism, contributing to or compounding the risk of neurological disability in this population.

We anticipate that preterm babies will have altered creatine homeostasis and reduced creatine accretion in the early postnatal period compared to an infant of the same postconceptional age. Whether or not relative creatine deficiency is associated with neurological deficit will be a secondary outcome of this study. As the results are mainly descriptive and a formal power calculation could not be completed, there is the possibility that sample size may become a limitation for some of the proposed analyses in this observational study. However, the data obtained will enable calculation of effect size for future studies. If our overall hypothesis is supported, this observational study will inform a future randomized control trial of infant dietary creatine supplementation following premature birth, with the aim of improving brain metabolism during a key stage of development and thereby reducing the burden of neurodevelopmental disability in this high-risk population.

## Author Contributions

MB, SE, HD, and DW conceptualized the study. MS is a research nurse coordinating recruitment, sample collection, and data management. AG was involved in ethics development and study design. GK, CB, DC, and RS developed the LC-MS/MS protocol for creatine and metabolite assessment. AS developed the MR data acquisition protocol. MD-T designed the statistical approaches to the study. MB and SE wrote the manuscript. All authors contributed to the drafting of the manuscript.

### Conflict of Interest Statement

The authors declare that the research was conducted in the absence of any commercial or financial relationships that could be construed as a potential conflict of interest.
